# Sandifer Syndrome: A Case Report

**DOI:** 10.31729/jnma.6472

**Published:** 2021-10-31

**Authors:** Abhigan Babu Shrestha, Prateet Rijal, Unnat Hamal Sapkota, Pashupati Pokharel, Sajina Shrestha

**Affiliations:** 1 M Abdur Rahim Medical College, Dinajpur, Bangladesh; 2 Maharajgunj Medical Campus, Institute of Medicine, Maharajgunj, Kathmandu, Nepal; 3 KIST Medical College, Imadol, Lalitpur, Nepal

**Keywords:** *gastroesophageal reflux*, *sandifer's syndrome*, *seizures*, *torticollis*

## Abstract

Sandifer syndrome is an extra oesophageal manifestation of gastrointestinal reflux disease that usually presents with torticollis and dystonia (often mimicking epilepsy). Here, we describe a case of a four and a half years old child with convulsion, neck contortion, and irritability. Gastrointestinal reflux disease was suspected on the earlier visit of the patient based on the presenting symptom of vomiting and cough. Electroencephalogram revealed normal findings. A barium meal radiograph was performed which was insignificant for gastrointestinal reflux disease and hiatal hernia. Complete blood count showed results suggestive of iron deficiency anaemia, while the rest of the biochemical parameters and the infection screening were normal. The case was confirmed by a medication trial for gastrointestinal reflux disease. This syndrome is often misdiagnosed as infantile seizure and musculoskeletal disorder. So, physicians need to have a sound knowledge of Sandifer Syndrome while assessing a child presenting with convulsion and torticollis.

## INTRODUCTION

Sandifer's syndrome (SS) is one of the rare complications of gastroesophageal reflux disease (GERD) that often presents with neurological manifestations such as, torticollis and dystonic body movements.^1^ There is very little medical documentation related to it so far. It has been estimated that approximately one case of SS occurs for every 100 children with symptomatic hiatal hernia.^2^ Several cases of SS without hiatal hernia, macroscopic esophagitis, or reflux symptoms, neural axis abnormalities have also been reported.^3^ Due to these varied and atypical presentations, multiple cases of missed or delayed diagnosis, and the incorrect intervention measures are common with SS.^4^

## CASE REPORT

**Figure 1:**
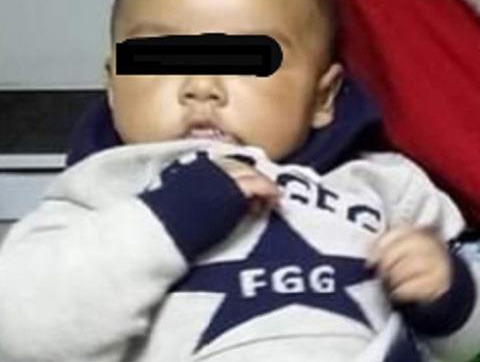
Torticollis (neck contortion) with irritability.

A four and half year male infant had attended our hospital with convulsion, neck contortion (torticollis), and stridor, and irritability (Figure 1).

There was a history of such an attack four times before. He was admitted before to this hospital with symptoms of bronchopneumonia and recurrent attacks of cold cough and vomiting. At that time with a history of vomiting, GERD was suspected. Barium meal chest X-ray was advised but not performed.

The milestones of motor and mental development were normal. His physical and neurological examinations were normal. There was no history of asphyxia and fever. The child was delivered normally at term. He was breastfed soon after birth and there was no history of physiological jaundice. There was no significant family history of such symptoms or any chronic illness.

EEG was done which revealed no electrical activities of the brain for the cause of convulsion. The blood panel showed mild microcytic hypochromic anaemia (suggestive of iron deficiency anaemia). Packed Cell Volume (PCV) = 32%, Mean Corpuscular Volume (MCV) = 73fL, Mean Corpuscular Haemoglobin (MCH) = 24pg and Haemoglobin= 10.5g/dl. Other biochemical parameters were all normal. Infection screening was normal. Barium meal X-ray was done which was insignificant for GERD and no evidence of hiatal hernia. Twenty-hour pH was not done due to lack of availability in our hospital.

Therefore, the diagnosis of Sandifer's syndrome was made based on typical clinical features and on response to medication trials. Barbiturate was prescribed first for a short duration of 14 days with tapering. Along with this for the management of GERD famotidine 1mg per kg body weight twice a day, domperidone 200mg per kg body weight thrice a day before a meal, omeprazole 1mg per kg body weight twice a day and an antacid suspension of aluminium oxide and magnesium hydroxide were prescribed for 14 days. After monitoring, the symptoms disappeared and barbiturate was taken off soon with a tapering dose. Famotidine and omeprazole were continued. Follow-up of the patient was done after a week then after 2 weeks. At the end of 3 weeks, the child recovered fully without any residual signs and symptoms.

## DISCUSSION

Sandifer syndrome is a relatively less common clinical syndrome that was first reported by Dr. M. Kinsbourne and named after neurologist Paul Sandifer. Children and adolescents are typically involved age group in this disease.^5^

It is an extra oesophageal manifestation of gastrointestinal reflux disease (GERD) that is sometimes associated with the hiatal hernia. The patient presents with crying, irritability, rumination, torticollis, dystonia, and hence might mimic epilepsy. Torticollis is atypical in the sense that there is no spasm of sternocleidomastoid and the positioning is intermittent with frequent changes of side. Dystonic movement usually spares limb with involvement of head, neck, back, and upper trunk. Epilepsy consists of rhythmic clonic components whereas Sandifer syndrome lacks.^4^

The exact pathophysiology of Sandifer syndrome is unknown. The torticollis and the dystonic movements occur in response to the pain associated with GERD in children. The dystonic movements are learned movements for temporary relief of the discomfort.^2^ It is suggested that neurological manifestation results due to vagal reflex with the vagal centre present in nucleus tractus solitarus.^6^ Sandifer syndrome may also present along with ocular manifestation, growth and developmental delay, irritability, and iron deficiency anaemia.^6^

Gastrointestinal symptoms such as abdominal pain, acid reflux, vomiting, and indigestion may be absent or poorly described due to children's young age.^4^ Thus if signs and symptoms are not properly evaluated then Sandifer syndrome may be incorrectly diagnosed as epilepsy, congenital torticollis, trauma, and various pathological conditions of head, neck, and CNS. This prompts unnecessary diagnostic procedures, misdiagnosis, and ultimately wrong treatment and financial burden to the family.^7^

It is now widely considered that more patients than currently reported might be suffering from Sandifer syndrome as these patients may be misdiagnosed as other neuromuscular or neuropsychiatric conditions.^4,8^ Proper evaluation of signs and symptoms is usually sufficient to diagnose Sandifer syndrome. Twenty-four-hour oesophageal pH measurement, upper gastrointestinal tract endoscopy, barium meal, chest X-ray Posterior-Anterior (PA) view, and scintigraphy studies can be done; but usually, 24 hr oesophageal pH is recommended as the first line of investigation when Sandifer syndrome is suspected.^6^

Sandifer syndrome can be managed by non-pharmacological, pharmacological, or surgical intervention. The non-pharmacological intervention involves recommendation for thickening of breast milk or formula by plain infant rice cereal or pre thickened formula, replacement of cow's milk by amino acid-based formula, placing the child upright after feeding, and avoidance of cigarette smoke.^4^’^9^ Pharmacological management is usually done via Proton Pump Inhibitor (Rabeprazole, Esomeprazole) or prokinetic agents (domperidone) or H2 receptor antagonist (Ranitidine, Famotidine).^10^ Surgical intervention via fundoplication is done only in case of the presence of hiatal hernia or medically refractory GERD.^4^

Sandifer syndrome has a good prognosis as appropriate management of GERD resolves other symptoms. No long-term complications or co-morbidities have to found to be associated with it.^5^ The healthcare workers need to have a sound knowledge of Sandifer Syndrome while assessing a child presenting with convulsion, neck contortion (torticollis), stridor, irritability, and dystonic episodes or atypical seizures. Sandifer syndrome has a good prognosis as the timely diagnosis and prompt treatment of GERD in patients with Sandifer Syndrome can help resolve further symptoms and other associated morbidity and largely contribute to improved patients' quality of life.

Our patient presented with convulsion, torticollis and stridor mimicking neurological pathology. With careful history, clinical examination and investigations we came up with the diagnosis of GERD manifesting as Sandifer Syndrome. Proper pharmacological treatment in the line of GERD subsided the neurological presentation of the patient. So, we recommend physicians to look for Sandifer's syndrome as well during the workup of a case of epilepsy in children.
